# Shock Processing of Amino Acids Leading to Complex Structures—Implications to the Origin of Life

**DOI:** 10.3390/molecules25235634

**Published:** 2020-11-30

**Authors:** Surendra V. Singh, Jayaram Vishakantaiah, Jaya K. Meka, Vijayan Sivaprahasam, Vijayanand Chandrasekaran, Rebecca Thombre, Vijay Thiruvenkatam, Ambresh Mallya, Balabhadrapatruni N. Rajasekhar, Mariyappan Muruganantham, Akshay Datey, Hugh Hill, Anil Bhardwaj, Gopalan Jagadeesh, Kalidevapura P. J. Reddy, Nigel J. Mason, Bhalamurugan Sivaraman

**Affiliations:** 1 Atomic Molecular and Optical Physics Division, Physical Research Laboratory, Ahmedabad 380009, India; surendra@prl.res.in (S.V.S.); jayakrishna@prl.res.in (J.K.M.); 2 Discipline of Physics, Indian Institute of Technology Gandhinagar, Gandhinagar 382355, India; 3 Solid State & Structural Chemistry Unit, Indian Institute of Science, Bangalore 560012, India; drjayaramv@gmail.com; 4 Planetary Science Division, Physical Research Laboratory, Ahmedabad 380009, India; vijayan@prl.res.in (V.S.); abhardwaj@prl.res.in (A.B.); 5 Department of Chemistry, Vellore Institute of Technology, Vellore 632014, India; vachandrasekaran@gmail.com; 6 Department of Biotechnology, Modern College of Arts, Science and Commerce, Pune 411005, India; rebecca.thombre@gmail.com; 7 Discipline of Biological Engineering, Indian Institute of Technology Gandhinagar, Gandhinagar 382355, India; vijay@iitgn.ac.in; 8 Centre for Nano Science and Engineering, Indian Institute of Science, Bangalore 560012, India; ambreshm@iisc.ac.in; 9 Atomic and Molecular Physics Division, BARC, Trombay, Mumbai 400085, India; bnrs@rrcat.gov.in; 10 Geosciences Division, Physical Research Laboratory, Ahmedabad 380009, India; vmmuruga@gmail.com; 11 Department of Aerospace Engineering, Indian Institute of Science, Bangalore 560012, India; akshay.datey@gmail.com (A.D.); jaggie@iisc.ac.in (G.J.); reddykpj@yahoo.com (K.P.J.R.); 12 Physical Sciences, International Space University, 67400 Illkirch-Graffenstaden, France; hugh.hill@isunet.edu; 13 School of Physical Sciences, University of Kent, Canterbury CT2 7NZ, UK

**Keywords:** shock processing, origin of life, astrobiology, amino acids, complex structures

## Abstract

The building blocks of life, amino acids, are believed to have been synthesized in the extreme conditions that prevail in space, starting from simple molecules containing hydrogen, carbon, oxygen and nitrogen. However, the fate and role of amino acids when they are subjected to similar processes largely remain unexplored. Here we report, for the first time, that shock processed amino acids tend to form complex agglomerate structures. Such structures are formed on timescales of about 2 ms due to impact induced shock heating and subsequent cooling. This discovery suggests that the building blocks of life could have self-assembled not just on Earth but on other planetary bodies as a result of impact events. Our study also provides further experimental evidence for the ‘threads’ observed in meteorites being due to assemblages of (bio)molecules arising from impact-induced shocks.

## 1. Introduction

The origin and evolution of life on Earth is one of the greatest unsolved mysteries in science. It is still unclear if life originated in “Darwin’s pool”, a hydrothermal vent, the ocean, a tidal pool or indeed elsewhere in the solar system [1]. The Miller–Urey (MU) experiment [2,3] provided direct experimental support for the “prebiotic soup” theory proposed by Oparin [4]. The results of MU experiments reported the chemical synthesis of organic compounds, including amino acids from simple gases probably synthesized by the Strecker pathway. The next step in evolution is proposed to be the formation of self-assembling complex organic molecules and polymers from simple amino acids [5]. The formation of peptides from amino acids is thus one of the crucial steps in the origin of life as peptides can form self-assembling molecular structures and interact with other classes of biomolecules, such as nucleic acids, lipids, etc. and enhance their structure and function [6]. Peptides are essential and are the structural building blocks that form supramolecular structures, such as helices, sheets, globules, fibers and tubes that can be used to build the basic architecture of a living cell [7]. Indeed, peptides form numerous structures useful for the cellular and molecular function of life due to different intermolecular interactions, such as electrostatic, hydrophobic, van der Waals and hydrogen bonding, etc. [7,8]. However, prebiotic availability of such structures was never explored.

Many studies have reported different abiotic mechanisms for peptide synthesis during prebiotic conditions, including synthesis in hydrothermal vents [9], irradiation [10,11,12], activation agents and oligomerization [1,5,13,14]. Impact shock events have also been responsible for the synthesis of organic matter that is presumed to be the prebiotic inventory of life on Earth [15]. Organics can also be produced by the shock processing of cometary gases [16]. The observation of large scale craters on the surface of the planetary bodies reminds us of the role of impact processes in planetary and lunar evolutions whilst many cometary bodies appear to be the result of collisions of constituent bodies. Such impacts release significant amounts of energy and, therefore, may provide pathways for large scale molecular synthesis.

Experiments have shown that shock processes, mimicking impact-induced shock events, on simple molecules lead to the synthesis of amino acids [17,18]. Recent studies have also reported the role of extraterrestrial impacts on the abiotic synthesis of amino acids [19,20] and peptides [5,21,22]. Molecular dynamics simulations have also shown that impact shock can drive the synthesis of complex organics like amino acids [23,24,25]. Further studies have reported survivability of amino acids under impact bombardment [26,27,28]. Along with amino acids, other biomolecules, such as nucleobases, sugars and amines are also known to be the product of impact driven processes [18,29,30,31]. Thus the role of impacts in prebiotic chemistry and formation of the first complex self-replicating macromolecules must be studied [1]. Blank et al. [21] used an impactor to process the amino acid ‘soup’ and observed peptide bonds to be present in the shocked solution. However, despite the recent impact induced formation of complex molecules, such as amino acids, its role in the origin of life is still unexplored. Therefore, it is imperative to subject amino acids to impact-induced shock conditions if we are to understand the next step in the evolution of life after the formation of complex molecules. 

In the present study, we have investigated the effect of impact by strong shock waves on amino acids in a shock tube. We exposed various amino acids to strong shock waves at temperatures of around 2500 K–8000 K. Our experimental conditions mimic a small portion (<10^4^ K) of the extreme conditions experienced in real impact events. To the best of our knowledge, this is the first report on the synthesis of complex structures by the impact of strong shock on amino acids.

## 2. Results and Discussion

### 2.1. Complex Structure Formation and Consequences for the Studies of the Origin of Life

**Figure 1:**
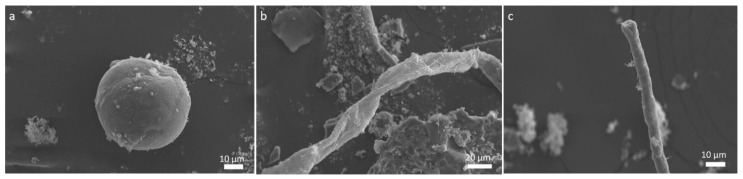
SEM images of the residues obtained after shock processing glycine. (**a**) globule structure, (**b**) fine filamentous thread feature and (**c**) cylindrical fibril feature.

**Figure 2:**
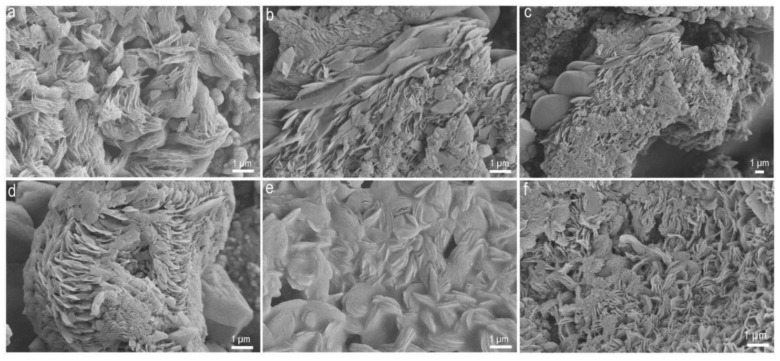
SEM images of residues obtained after shock processing of two amino acid mixtures (Table S1). (**a**–**c**) Short feather like features, (**d**) shows the formation of ordered scale features in a glycine-glutamic acid mixture, (**e**) rose petal like features in an asparagine–glutamic acid mixture and (**f**) short thread like features in a glycine-glutamic acid mixture.

Single amino acids, such as glycine, a combination of two amino acid mixtures, four amino acid mixtures and 18 amino acid mixtures were exposed to impact-induced shock using a shock tube. We started with the simplest amino acid, glycine, and further combinations of two and four amino acids were chosen on the basis of positively charged, negatively charged and neutral amino acids as suggested by Miller and Orgel [32]. The eighteen amino acids were used from the 20 standard amino acids. The various operational parameters and experimental shock conditions, such as the Mach number, the reflected shock temperature (estimated) and the pressure conditions are listed in Table S1. Increasing the number of amino acids in the mixture (Table S1), in equal proportions weight percentage ratio (w/w), resulted in a shocked sample with a sticky consistency and the structures observed by scanning electron microscopy (SEM) were solid clumps along with twisted and a few cylindrical threads like helices. No structures were observed in SEM imaging of the unshocked sample (control: Figure S5a,b). This indicates that shock processing of amino acids leads to the synthesis of complex macrostructures. The shocked sample of pure glycine revealed the formation of a distinct globule of ~ 45 to 50 μm diameter, with a smooth texture and a spongy appearance (Figure 1). Interestingly, a ribbon/thread structure similar to filamentous peptide fibrils [33], stretching to a few tenths of a micron, was also observed (Figure 1). Adding another amino acid, glutamic acid, in equal weight proportions with glycine, shows the formation of an entirely different macrostructure in the shocked sample (Figure 2). The structures formed in Figure 2 resemble hierarchical ordered floral structures viz. rose flower petals and shorter bunched threads. Similar hierarchically ordered structures, floral structures formed by diphenylalanine peptide, have been reported earlier with peony-like flower morphology [34]. Similarly, the shock processed mixture of asparagine and glutamic acid samples presented different complex microstructures (Figure 2e). 

**Figure 3:**
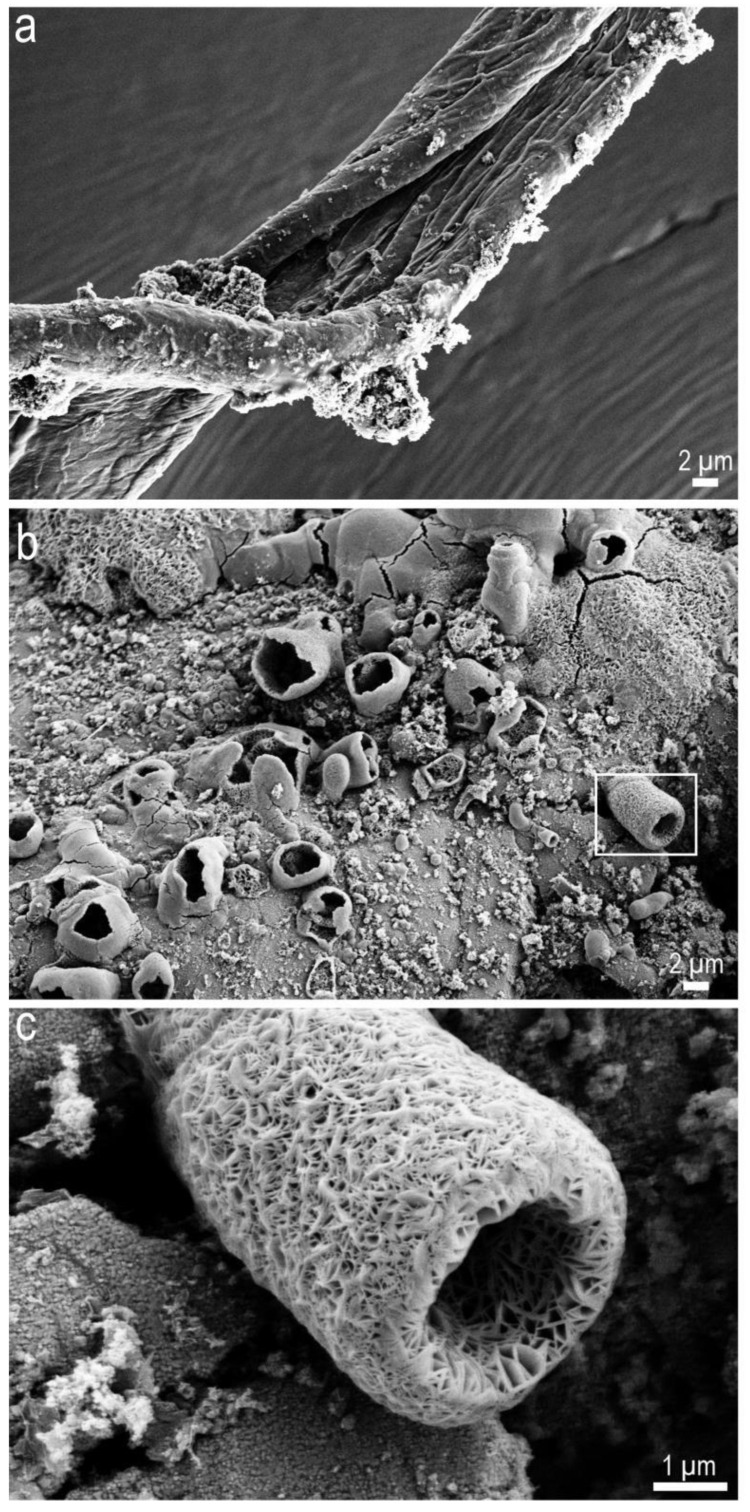
SEM images of residues obtained after shock processing of equal proportions of four amino acids (Table S1). (**a**) Thick thread feature containing fine threads running all along, (**b**) shows many porous features and (**c**) the porous cylindrical feature.

Further experiments performed with the addition of two more amino acids, to create a mixture of four amino acids, namely lysine, aspartic acid, arginine and glutamic acid (Table S1) in equal weight proportions, resulted in a dark black sticky residue. SEM imaging of this last sample showed threads (Figure 3) and, a much more surprising result, the formation of a porous cylindrical structure (Figure 3), a few microns in diameter. Porous and cavity structures are vital in the assembly and proper functioning of proteins [35]. Additionally, such porous and multi-chambered structures are feasible candidates for primitive abiotic cellularity due to their energy capture and conversion capacity [36].

**Figure 4:**
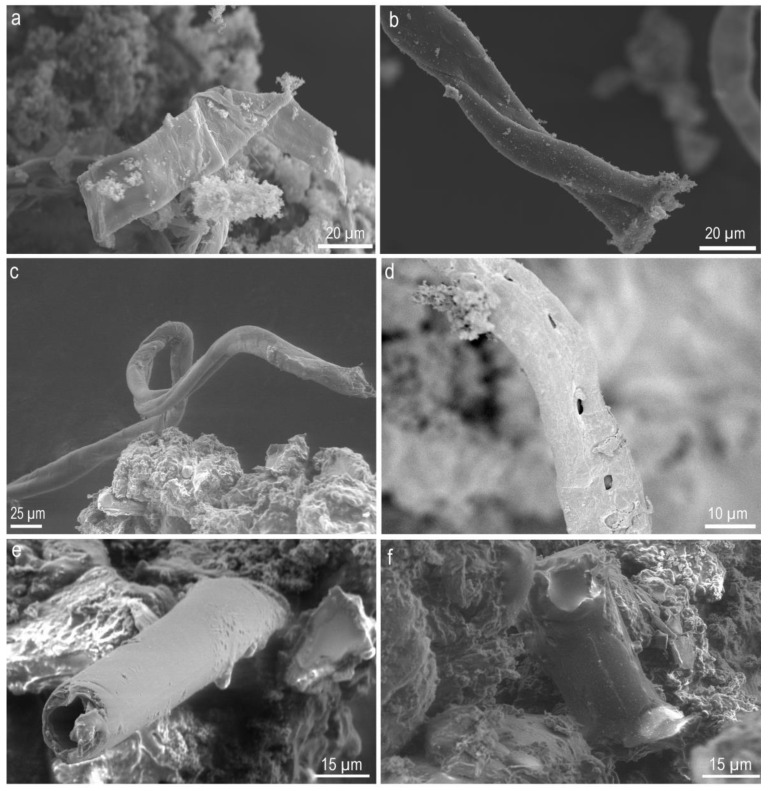
SEM images of the residue obtained after shock processing of 18 amino acids (Table S1). Several features, including (**a**) a thin ribbon, (**b**) a typical helical structure, (**c**) a branching filamentous thread and (**d**–**f**) a hollow tubule, were observed.

By further increasing the number of amino acids to eighteen in the mixture (Table S1), mixed in equal weight proportion, a thick black sticky residue resulted and many different structures were observed, including threads and ribbons as well as twisted and cylindrical (Figure 4). Upon closer inspection, we could clearly see that the threads were made of small (about a micron size) features. By reducing the shock temperature to 4000 K–5000 K (Table S1), the number of threads formed was found to increase. The length of the threads formed was quite surprising as they spanned more than one mm (Figure S7). Formation of such long-range ordered structures from basic building blocks is of crucial importance to complex biological systems with multiple functional properties [37]. The twisted threads were observed to split (Figure 4), which is an indication of an even more complex structure. Most of the threads were observed to be solid; nevertheless, our visual inspections suggest tubular structures were also found (Figure 4). So a variety of structures were observed when many amino acids were mixed and subjected to extreme shock conditions. As different amino acids possess different physicochemical properties, depending upon their size, charge and polarity of the side chain, etc., they can self-assemble into various structures depending upon the amino acid sequence [7].

Further, transmission electron microscopy (TEM) analysis of the shocking amino acids revealed membrane like structures with branching features being clearly observed with fine threads running throughout (Figure S8). Hence an amazing variety of structures were observed when a mixture of many amino acids was subjected to extreme shock conditions. Overall, the effect of impact on amino acids resulted in residues that demonstrated the formation of structures that resembled supramolecular cellular structures, such as fibrils, α-helical peptides, thread like microfibrils, hierarchical ordered floral structures and self-assembled hollow nanotubes [7,33,34,38].

In nature, filamentous proteins, such as actin polymers, microtubules, etc. are composed of monomeric building blocks. These nanosized peptides assemble spontaneously or by other chemical attraction or bond formation processes to form fibers and filaments that are a few micrometers in length [33]. These characteristic structures can be observed using electron microscopy [39,40]. Jia & Kuruma 2019 [39] have stated the wide application of state of the art imaging techniques in the interpretation of various self-assembling systems and thus it could be a useful tool in the origin of life research.

Further IR spectroscopy of shock processed residues from different mixtures of amino acids show the presence of an amide I band [41], a signature of peptide bonds, along with amino acid signatures (Figure S6). This gives us a clue that the various complex structures could be the outcome of peptide assemblies as it is well known that peptides are masters of self-assembly and are known to form a variety of structures [7,8].

Nonetheless, such a rich abundance of structures revealed by microscopic technique seem to achieve the combination of two basic characteristics in the context of life i.e., structural order and complexity [42]. It has already been shown that vesicle membrane like structures may be formed by exposure of irradiated prebiotic compounds to water [43]. Electrostatic interactions induced by short, positively charged, hydrophobic peptides may then attach RNA to vesicle membranes and thus the first forms of life may have been simple cells containing systems of peptides and short strands of nucleic acid, such as RNA.

### 2.2. Implications to the Structure Observed in Meteorites

**Figure 5:**
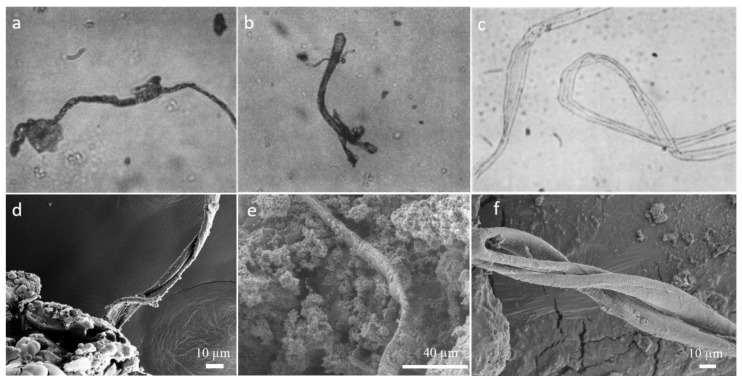
Photomicrographs of filamentous structures observed of size range ~ 20 µm in (**a**) Mighei meteorite, (**b**) Murray meteorite, (**c**) Dimmit meteorite [45] and (**d**–**f**) similar structures observed in SEM micrographs of shock processed residue of amino acid.

**Figure 6:**
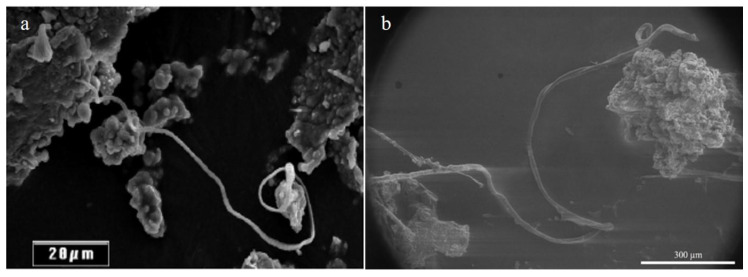
(**a**) A long helical coil observed in the Orgueil meteorite [54]. (**b**) A similar long thread seen in shock processing of amino acids.

The various features reported in our studies have a striking resemblance to microstructures observed in some meteorites [44,45]. These microstructures were titled as “organized elements” by Claus and Nagy, 1961 [44], which resembled biological forms but their biogenic origin was unknown and they were also excluded as being of terrestrial contamination [46]. However, existence of such structures in meteorites was discussed in detail and no convincing evidence was found concerning their origin [47,48,49,50]. Further studies identified these structures as microfossil remains [44,51,52]. Hoover et al. [53] and Hoover 2011 [54], also deciphered these elements as microfossils of extra-terrestrial life forms, indigenous to the meteorite, by comparing these structures with living and fossilized cyanobacteria. However, our results on the creation of microstructures in shock processed amino acids give a more plausible explanation for the formation of these structures in meteorites when they are subjected to impact induced shock events. A comparison of the structures we have observed with similar structures in several meteorites is shown in Figure 5 and Figure 6 as well as Supplementary Figures S9–S11. The striking similarities between the two suggest that shock induced processing of amino acids, known to be present in meteorites, can lead to the formation of microstructures.

## 3. Materials and Methods

**Figure 7:**
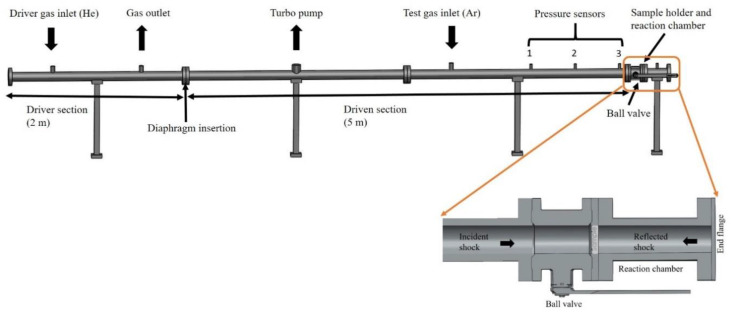
Schematic diagram of Material Shock Tube (MST1).

The shock tubes used in the current research are the Material Shock Tube (MST1), (Biennier et al. 2017, [55]) in the Department of Solid State and Structural Chemistry Unit, Indian Institute of Science (Bangalore, India), and the High-Intensity Shock Tube for Astrochemistry (HISTA) in the Physical Research Laboratory (Ahmedabad, India). Both shock tubes are similar in their construction and the instrumentation used; therefore, experiments were carried out and repeated at both the shock tube facilities. The shock tube of 80 mm inner diameter consists of a 2 m long driver section and a 5 m long driven section separated by a metallic diaphragm (Figure 7). Generally, aluminum diaphragms (Figure S1), up to about 2 mm thickness, are used with appropriate grooves to guide the proper bursting pressure to produce shock waves of the required strength. After sterilizing the inner surface of the shock tube using pure acetone/isopropyl alcohol/ethanol solution to avoid any contamination, a diaphragm of the desired thickness is placed between the driver section and the driven section. To obtain different reflected shock temperatures, diaphragms of varying thickness are used. The sample holder for the study of shock wave interactions with test samples is attached to the end of the shock tube through a manually operated gate valve. The amino acid sample is uniformly distributed over the sample holder (Figure S2) parallel to the flow of gas inside the shock tube and the interaction of strong shock heated gas with the sample occurs at the end flange of the 300 mm long reaction chamber (Figure S3). The sample experiences a reflected shock pressure of 12–34 bar and a temperature of 2500 K–8000 K (estimated) with a Mach number of 2–6, for a 1–2 ms duration in the reaction chamber as listed in Table S1. 

The amino acid mixture/mixtures (Tables S1 and S2) are uniformly deposited for each experiment over the sample holder (Figure S2). The driven section of the shock tube is purged 2–3 times with ultra-high pure Argon (99.999%) which removes residual gas impurities and then the driven section is pumped to a high vacuum, up to 2 × 10^−4^ mbar, using a turbo molecular pumping system. Evacuation of the shock tube is performed slowly to prevent any dispersal of the sample inside the chamber. The driven section is filled with ultra-high pure Argon (99.999%) up to the desired pressure (Table S1). The driver section is also pumped to a rotary vacuum, up to 0.01 bar, and is then rapidly filled with high-pressure helium gas at a very high mass flow rate until the diaphragm ruptures. The sudden rupture of the diaphragm generates a shock wave that travels through the driven section and is reflected from the end flange of the driven section. The ball valve next to the reaction chamber is closed immediately after the rupture of the diaphragm. The high pressure inside the reaction chamber is brought to equilibrium by slowly exhausting into the atmosphere. The solid residue left in the reaction chamber was collected and stored under inert conditions for further analysis (Figure S3).

Three pressure sensors (Model 113B22, PCB Piezotronics, NY, USA) surface mounted at three different locations on the driven section, used to obtain a pressure signal, are recorded with the help of Tektronix digital storage oscilloscope (Model TDS2014B, Tektronix Inc, MS, USA). The shock speed (*V_s_*) and Mach number (*M_s_*) are calculated by finding the time is taken (∆*t*) by the shock wave to travel cross the distance (∆*x*) of two pressure sensors, with the help of the recorded pressure signal. The primary and reflected shock pressure signals recorded using the digital storage oscilloscope are shown in Figure S4. The temperature behind the reflected shock wave is calculated using the following one-dimensional normal shock equations known as Rankine-Hugoniot relations [56],

(1) $$\;V_{s}=\frac{∆x}{∆t};M_{s}=\frac{V_{s}}{a}=\frac{V_{s}}{\surd \gamma RT_{1}}$$

(2) $$\frac{T_{5}}{T_{1}}=\frac{\left[2\left(\gamma -1\right)M_{s}^{2}+\left(3-\gamma \right)\right]\left[\left(3\gamma -1\right)M_{s}^{2}-2\left(\gamma -1\right)\right]}{{\left(\gamma +1\right)}^{2}M_{s}^{2}}$$

where *γ* is the specific heat ratio of the test gas argon, R is the universal gas constant, a is the speed of sound in the test gas argon and *T*_1_ is the ambient temperature of the test gas. The reflected shock temperature *T*_5_ is a function of the shock Mach number *M_S_* and *γ*, as given by the Equation (2). The calculated values of the shock Mach numbers and the reflected shock temperatures (*T*_5_) and recorded reflected shock pressures (P_5_) for each experiment are listed in Table S1.

## 4. Conclusions

In the present study, we have investigated the effect of strong shock impact at extreme thermodynamic conditions on several amino acids and their mixtures, which provides insight into the role of impact events in prebiotic evolution. This is the first reported case on the formation of complex macroscale structures from the shock processing of simple amino acids. Scanning electron microscopic analysis provided valuable insights on the self-assembled structures that showed structural complexity from nanometer to millimeter length scales. These structures resembled supramolecular cellular structures, such as fibrils, α-helical peptides, thread like microfibrils, hierarchical ordered floral structures and self-assembled hollow nanotubes. Furthermore, microstructure analysis of the shock processed organics using electron microscopy revealed hierarchical formation and assembly of molecular structures that may be produced in impact shock to meteorites and may explain some of the structures revealed in some meteorite samples in response to impact shock in meteorites. In future endeavors, we expect this research work will expand towards the formation of more complex structures which are closer to biological architectures, not only in structural resemblance but also in performance, by considering the role of other functional biomolecules, such as nucleobases, fatty acids, nucleic acids, etc. The developed method will also provide a novel application of shock tubes in biomaterial synthesis to design and engineer a superstructure with long-range order from simple building blocks.

## Supplementary Materials

The following are available online. Supplementary materials file 1: Figures S1–S4: Experimental details, Figure S5: SEM micrographs of unprocessed amino acids, Figure S6: IR spectra of shock processed residue, Figures S7 and S8: SEM and TEM micrographs of shock processed residue, Table S1: Summarized the experimental parameters and the estimated shock temperature, Table S2: List of amino acids used in the experiments. Supplementary materials file 2: Figures S9–S11: Visual comparison of microstructures observed in meteorite samples with structures observed in shock processed amino acid mixtures.
